# CXCL9 Associated with Sustained Virological Response in Chronic Hepatitis B Patients Receiving Peginterferon Alfa-2a Therapy: A Pilot Study

**DOI:** 10.1371/journal.pone.0076798

**Published:** 2013-10-04

**Authors:** I-Cheng Lee, Yi-Hsiang Huang, Chien-Wei Su, Yuan-Jen Wang, Teh-Ia Huo, Kuei-Chuan Lee, Han-Chieh Lin

**Affiliations:** 1 Division of Gastroenterology, Department of Medicine, Taipei Veterans General Hospital, Taipei, Taiwan; 2 Health Care Center, Taipei Veterans General Hospital, Taipei, Taiwan; 3 Institute of Clinical Medicine, National Yang-Ming University School of Medicine, Taipei, Taiwan; 4 Institute of Pharmacology, National Yang-Ming University School of Medicine, Taipei, Taiwan; 5 Department of Medicine, National Yang-Ming University Hospital, I-Lan, Taiwan; Duke University, United States of America

## Abstract

**Background and Aims:**

There is lack of a practical biomarker to predict sustained virological response (SVR) in chronic hepatitis B (CHB) patients undergoing peginterferon alfa-2a (PEG-IFN). The aim of this pilot study was to identify immunological features associated with SVR.

**Methods:**

Consecutive 74 CHB patients receiving 24 weeks (for hepatitis B e antigen (HBeAg)-positive) or 48 weeks (for HBeAg-negative) PEG-IFN, were prospectively enrolled. Serum HBV viral loads, hepatitis B surface antigen (HBsAg), CXCL9, IFN-γ-inducible protein 10 (IP-10), interferon-gamma (IFN-γ) and transforming growth factor beta (TGF-β) were measured at baseline and week 12. SVR was defined as HBeAg seroconversion combined with viral load <2000 IU/mL in HBeAg-positive (n=36), and viral load <2000 IU/mL in HBeAg-negative patients (n=38) at 48 weeks after the end of treatment.

**Results:**

Nineteen patients (25.7%), 7 in HBeAg-positive and 12 in HBeAg-negative, achieved SVR. There were significant declines of HBV DNA, HBsAg, IP-10 and IFN-γ levels at week 12. In multivariate analysis, pre-treatment CXCL9 >80 pg/mL, HBV DNA <2.5 x 10^7^ IU/mL and on-treatment HBV viral load, HBsAg decline >10% at week 12 were predictors of SVR. The performance of CXCL9 in predicting SVR was good in patients with HBV DNA <2.5 x 10^7^ IU/mL, particularly in HBeAg-negative CHB cases (positive predictive value, PPV= 64.3%).

**Conclusions:**

Pre-treatment CXCL9 level has the potential to select CHB patients who can respond to PEG-IFN, especially in HBeAg-negative patients with low viral loads.

## Introduction

Chronic hepatitis B virus (HBV) infection remains a challenging global health problem, with more than 350 million carriers worldwide [1]. The spectrum and the natural course of chronic HBV infection are diverse and variable, ranging from an inactive carrier state to progressive chronic hepatitis B (CHB), which may evolve to hepatic decompensation, cirrhosis and hepatocellular carcinoma (HCC) [2]. Both host and viral factors play critical roles in the natural history of CHB, disease activity as well as efficacies of antiviral therapies [3,4]. Impaired host immune response against HBV is commonly observed in patients with CHB, which might be related to persistent high viral loads with subsequent T cell exhaustion [5]. Current antiviral strategies for CHB aim at effective viral suppression as well as restoration of HBV-specific immune responses.

Peginterferon-alpha-2a (PEG-IFN), which has direct antiviral and immuno-modulatory effects, is currently one of the first-line treatment options for CHB [6,7]. Antiviral therapy with PEG-IFN has been shown to be effective in suppressing HBV replication, and may result in hepatitis B e antigen (HBeAg) seroconversion, hepatitis B surface antigen (HBsAg) clearance, normalization of alanine aminotransferase (ALT) levels, and histological improvement [6,7]. Patients who achieve an IFN-induced virological response may have a long-term therapeutic effect and a reduced risk of cirrhosis and HCC [8,9]. However, only about 30% of CHB patients treated with PEG-IFN could achieve a sustained virological response (SVR) [10–13]. Several baseline and on-treatment indicators have been identified to predict treatment response to PEG-IFN [7,10]. In HBeAg-positive CHB, low viral load, high serum ALT levels, HBV genotype and high activity scores on liver biopsy are pre-treatment predictors of HBeAg seroconversion [14,15], whereas currently there are no strong pre-treatment predictors of virological response in HBeAg-negative CHB patients [7]. During the treatment, declines of viral load and HBsAg levels at 12 weeks are strong predictors of virological response in both HBeAg-positive and HBeAg-negative patients [16–18].

Based on the immuno-modulatory properties of PEG-IFN, host immune status may have influence on the efficacy of PEG-IFN for CHB. In recent years, several studies have shown the potential roles of cytokines and chemokines in chronic viral hepatitis. CXCL9 (monokine induced by IFN-γ [MIG]) and IP-10 (IFN-γ-inducible protein 10, also called CXCL10) have been reported to play important roles during hepatitis flares in CHB [19]. In patients with hepatitis C virus (HCV) infection, lower baseline and on-treatment IP-10 levels may predict a higher rate of virological response to IFN-based therapy [20–22]. Recently, we also demonstrated a correlation of IP-10 with higher hepatitis activity in patients with CHB [4]. Pro-inflammatory cytokines interferon-gamma (IFN-γ) and transforming growth factor beta (TGF-β) may also have roles in suppressing HBV replication [1,23,24]. However, the clinical significance of these cytokines and chemokines during PEG-IFN therapy is unclear. IL28B single nucleotide polymorphisms (SNPs) have been shown to correlate with IFN-induced treatment response in patients with chronic hepatitis C, as well as HBeAg seroconversion in HBeAg-positive CHB patients [25–28]. However, the role of IL28B SNPs in predicting SVR to PEG-IFN therapy for CHB patients is still debated.

Currently, pre-treatment and on-treatment predictors of SVR in CHB patients under PEG-IFN therapy remain unsatisfactory to meet clinical need. The aim of this pilot study was to investigate the dynamics of cytokines and chemokines during PEG-IFN treatment, and to evaluate the baseline and on-treatment immunological and viral factors associated with SVR in CHB patients treated with PEG-IFN.

## Materials and Methods

### Patients

From January 2010 to July 2011, consecutive 74 CHB patients receiving PEG-IFN alfa-2a 180µg per week at the Taipei Veterans General Hospital were prospectively enrolled. All patients were positive for serum HBsAg for more than 6 months and patients receiving PEG-IFN therapy fulfilled the treatment criteria for CHB according to the American Association for the Study of Liver Disease (AASLD) treatment guidelines, i.e. serum ALT levels >80 U/L [2 x upper limit of normal (ULN)] with HBV DNA >20,000 IU/mL in HBeAg-positive patients or >2,000 IU/mL in HBeAg-negative patients [6]. All patients were negative for any of the following points: (1) coinfection with HCV, hepatitis D virus, or human immunodeficiency virus, (2) alcoholic liver disease, (3) suspected autoimmune diseases with antinuclear antibody (ANA) titer ≥1:160, positive test for anti-smooth muscle antibody or anti-mitochondrial antibody, (4) use of hepatotoxic drug or Chinese herb, and (5) radiological evidence of cirrhosis or HCC (i.e., abdominal sonogram, computed tomography scan, or magnetic resonance imaging scans). In general, the treatment duration was 24 weeks for HBeAg-positive, and 48 weeks for HBeAg-negative cases, which is under the regulation of Bureau of National Health Insurance, Taiwan. In HBeAg-positive patients, additional 24 weeks of PEG-IFN was allowed without reimbursement.

After initiating PEG-IFN treatment, patients were subsequently followed at the outpatient clinic every 2 to 4 weeks. Peripheral blood samples and HBV viral loads were evaluated at baseline, week 12, the end of treatment (EOT), 24 and 48 weeks after PEG-IFN treatment for serological and virological tests. This study was approved by the Institutional Review Board, Taipei Veterans General Hospital, which complied with standards of the Declaration of Helsinki and current ethical guidelines. All patients provided written informed consents for participation of the study.

### End point and definition

The primary end point was sustained virological response (SVR). In HBeAg-positive patients, SVR was defined as a combination of HBeAg seroconversion, indicated as seronegative of HBeAg and seropositive of anti-HBe in serum, with HBV DNA level less than 2000 IU/mL at 48 weeks after the end of PEG-IFN therapy. In HBeAg-negative patients, SVR was defined as HBV DNA level less than 2000 IU/mL at 48 weeks after the end of PEG-IFN therapy [7].

### Liver biochemistry and viral serology tests

Serum biochemical studies were performed using a systemic multi-autoanalyzer (Technicon SMAC, Technicon Instruments Corp., Tarrytown, NY). The serum samples were tested for the presence of HBeAg and anti-HBe antibody using radio-immunoassay (Abott Laboratories, North Chicago, IL), while HBV DNA was determined by Roche Cobas Tagman HBV DNA assay (detection limit of 12 IU/mL, Roche Diagnostics, Switzerland).

### Detection, genotyping and sequencing of HBV DNA

Genotyping of HBV was performed by PCR restriction fragment length polymorphism (PCR-RFLP) of the surface gene of HBV [29,30]. Briefly, DNA was extracted from serum, and the fragment of the HBV genome between nucleotide position 120 and 604 was amplified by semi-nested PCR. The PCR products were subsequently treated with restriction enzymes. After incubation, the samples were run on a 4% agarose gel and stained by ethidium bromide. To confirm the correct genotyping, direct sequencing from the PCR products was done.

To detect precore G1896A and basal core promoter (BCP) A1762T/G1764A mutations, sequencing of the core region of HBV DNA was performed in all patients. Semi-nested PCR was performed by using a pair of primers: internal primers 1653F (5’-CATAAGAGGACTCTTGGACT-3’, position 1653-1672) and 1974R (5’-GGAAAGAAGTCAGAAGGC-3’, position 1974-1957); external primers: 1623F (5’-TCGCATGGAGACCACCGTCT-3’, position 1623-1640) and 2076R (5’-ATAGCTTGCCTGAGTGC-3’, position 2076-2060) as previously described [6,21].

The PCR products were then subjected to the dye-terminator cycle sequencing reaction using specific primers according to the standard protocol provided by the manufacturer (Dye terminator cycle sequencing core kit no. 402117, Perkin Elmer Cetus Corp., Norwalk, CT). To avoid false positive results, instructions to prevent cross contaminations were strictly followed.

### Serum HBsAg quantification

HBsAg levels were quantified using the Elecsys HBsAg II assay (Roche Diagnostics GmbH, Mannheim, Germany) according to the manufacturer’s instructions. The detection limit of the Elecsys HBsAg II assay was 0.05 IU/mL.

### Enzyme-linked sorbent assay (ELISA)

The concentrations of CXCL9 and IP-10 were tested by commercialized human cytokine ELISA kits from PeproTech (Rocky Hill, NJ). The concentrations of IFN-γ and TGF-β were tested by commercialized human cytokine ELISA kits from eBioscience (San Diego, CA). The procedure followed the instruction provided by manufactures.

### IL28B genotyping

Four SNPs of IL28B including rs8105790, rs12979860, rs8099917 and rs10853728 were chosen according to previous reports and our study [4,25–28,31]. The genotype of rs12979860 was tested using TaqMan custom-designed rs12979860 probes (Applied Biosystems, Foster City, CA; forward primer GCCTGTCGTGTACTGAACCA, reverse primer GCGCGGAGTGCAATTCAAC, and the probes TGGTTCGCGCCTTC [VIC] and CTGGTTCACGCCTTC [FAM], respectively) [32]. The genotypes of the rs8105790, rs8099917 and rs10853728 were determined with the ABI TaqMan SNP genotyping assays (Applied Biosystems) and with predesigned commercial genotyping assays (ABI assay C__43813808_10, C__11710096_10, C__11710090_10). Briefly, PCR primers and two allelic-specific probes was designed to detect a specific SNP target. The PCR reactions were performed in 96-well microplates with ABI 7900 real-time PCR (Applied Biosystems) as previously described [4].

### Statistical analyses

All statistical analyses were performed using the Statistical Package for Social Sciences (SPSS 17.0 for Windows, SPSS Inc, Chicago, IL). Values were expressed as median (ranges) or as mean ± standard deviation when appropriate. The correlation between serum ALT and chemokine levels were tested by Spearman’s test. Pearson chi-square analysis or Fisher exact test was used to compare categorical variables, while the Student t test or Mann-Whitney U test was used to compare continuous variables. Wilcoxon signed ranks test was used to compare serial changes in virological and immunological factors. Variables with p<0.1 were analyzed by multivariate logistic regression analysis to identify independent variables for predicting SVR. The best cut-off value of each variable was determined by the receiver-operating characteristic (ROC) curve (Youden index) using MedCalc (version 4.20, MedCalc Software, Mariakerke, Belgium). A 2-tailed p value <0.05 was considered statistically significant.

## Results

### Characteristics of CHB patients receiving PEG-IFN therapy

Baseline characteristics of the 74 CHB patients were summarized in Table 1. Patients were predominantly males (77%) and genotype B (64.9%). BCP mutations and precore mutations were observed in 31.1% and 47.3% of patients, respectively. The prevalence of the major IL28B genotypes rs8105790 TT, rs12979860 CC, rs8099917 TT, and rs10853728 CC were 94.6%, 93.2%, 94.6% and 71.6%, respectively. In the 36 HBeAg-positive patients, 30 (83.3%) received 24-weeks and 5 6 (16.7%) received 48-week PEG-IFN therapy. All 38 HBeAg-negative patients received 48-week PEG-IFN therapy. Seventy-three (98.6%) patients completed the full course of PEG-IFN therapy, while 1 (1.4%) patient discontinued PEG-IFN treatment due to intolerance and were switched to oral antiviral therapies. The patient who failed to complete PEG-IFN therapy was classified as non-responder to PEG-IFN for analysis in this study.

**Table 1:** Baseline characteristics of the CHB patients with or without sustained virological response (SVR).

	All patients n = 74	No SVR n = 55 (74.3%)	SVR n = 19 (25.7%)	*p*
Age (years)	41.6 ± 10.2	40.5 ± 10.0	44 ± 11	0.119
Male sex, n (%)	57 (77.0)	42 (76.4)	15 (78.9)	1.000
Treatment duration				0.514
24 weeks	30 (40.5)	24 (43.6)	6 (31.6)	
48 weeks	44 (59.5)	31 (56.4)	13 (68.4)	
HBeAg-positive, n (%)	36 (48.6)	29 (52.7)	7 (36.8)	0.353
Genotype				0.922
B, n (%)	48 (64.9)	35 (63.6)	13 (68.4)	
C, n (%)	26 (35.1)	20 (36.4)	6 (31.6)	
BCP mutation, n (%)	23 (31.1)	18 (32.7)	5 (26.3)	0.816
Precore mutation, n (%)	35 (47.3)	23 (41.8)	12 (63.2)	0.180
*IL28B* polymorphisms				
rs8105790 TT/CT/CC, n (%)	70/4/0 (94.6/5.4/0)	51/4/0 (92.7/7.3/0)	19/0/0 (100/0/0)	0.353
rs12979860 CC/CT/TT, n (%)	69/5/0 (93.2/6.8/0)	50/5/0 (90.9/9.1/0)	19/0/0 (100/0/0)	0.319
rs8099917 TT/GT/GG, n (%)	70/4/0 (94.6/5.4/0)	51/4/0 (92.7/7.3/0)	19/0/0 (100/0/0)	0.567
rs10853728 CC/CG/GG, n (%)	53/20/1 (71.6/27/1.4)	39/15/1 (70.9/27.3/1.8)	14/5/0 (73.7/26.3/0)	1.000
ALT (U/L)	216 ± 200	202 ± 180	258 ± 251	0.276
AST (U/L)	110 ± 98	99 ± 78	144 ± 139	0.205
HBV DNA (Log_10_ IU/mL)	7.16 (3.36-8.04)	7.34 (3.55-8.04)	5.82 (3.36-8.04)	0.004
HBsAg (Log_10_ IU/mL)	3.49 (1.45-5.46)	3.64 (1.53-5.46)	3.14 (1.45-5.27)	0.028
CXCL9 (pg/mL)	65.1 (12.3-1000)	45.3 (12.3-1000)	191.7 (22.0-1000)	0.002
IP-10 (pg/mL)	89.6 ± 56.8	101.7 ± 55.2	106.1 ± 62.7	0.833
IFN-γ (pg/mL)	30.2 (undetectable-1416)	32.7 (undetectable-1416)	29.6 (undetectable-886)	0.420
TGF-β (pg/mL)	939 ± 503	903 ± 537	1040 ± 388	0.232

### Treatment response

Overall, 19 patients (25.7%) had achieved SVR at 48 weeks after the end of PEG-IFN treatment. Among the 36 HBeAg-positive patients, 12 (33.3%) achieved HBeAg seroconversion, including 10 of the 30 patients (33.3%) receiving 24-week course of treatment and 2 of the 6 patients (33.3%) receiving 48-week course of treatment; whereas only 7 (19.4%) finally achieved SVR, including 6 of the 30 patients (20%) receiving 24-week course of treatment and 1 of the 6 patients (16.7%) receiving 48-week course of treatment. In HBeAg-negative patients, 12 (31.6%) achieved SVR and 2 cases (5.3%) achieved HBsAg clearance at 48 weeks after the end of PEG-IFN treatment. During the extended follow-up period between 24 to 48 weeks post treatment, one HBeAg-positive patient developed HBeAg seroconversion and one HBeAg-negative patient had viral relapse.

### Dynamic changes of virological and immunological factors

Dynamic changes of serum HBV DNA, HBsAg, cytokine and chemokine levels from baseline to week 12 of PEG-IFN treatment were shown in Table 2. Viral load, HBsAg, IP-10 and IFN-γ levels significant declined after 12 weeks of PEG-IFN treatment. No significant changes were observed in CXCL9 and TGF-β levels from baseline to week 12 in overall patients.

**Table 2:** Dynamic changes of HBV viral load, HBsAg and cytokine/chemokine levels in CHB patients at baseline and week 12 of PEG-IFN therapy.

	Baseline	Week 12	*p* *
HBV DNA, Log_10_ IU/mL	7.16 (3.36-8.04)	4.12 (undetectable -8.04)	<0.001
HBsAg, Log_10_ IU/mL	3.49 (1.45-5.46)	3.43 (0.89-4.93)	<0.001
CXCL9, pg/mL	65.1 (12.3-1000)	52.1 (1.7-1000)	0.147
IP-10, pg/mL	89.6 ± 56.8	73.4 ± 95.0	<0.001
IFN-γ, pg/mL	30.2 (undetectable-1416)	16.8 (undetectable-1245)	0.003
TGF-β, pg/mL	939 ± 503	1003 ± 463	0.243

* By Wilcoxon signed ranks test**.**

**Figure 1:**
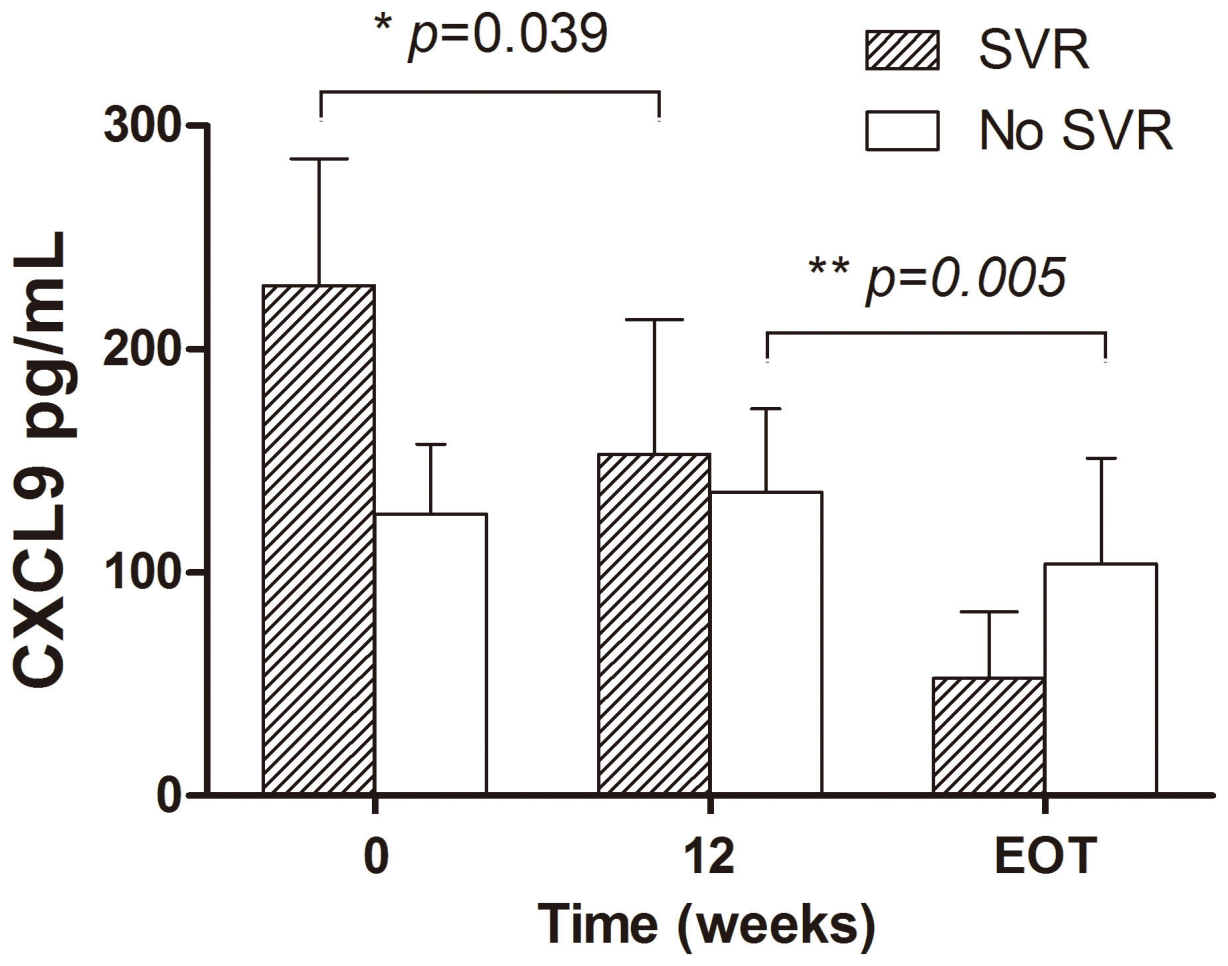
Kinetics of serum CXCL9 levels during PEG-IFN treatment in CHB patients with or without SVR. In patients with SVR, CXCL9 levels significantly decreased from baseline to week 12, whereas in patients without SVR, CXCL9 levels remained high at week 12 but decreased later from week 12 to EOT. Data were presented as mean ± standard errors of the means (SEM). EOT: end of treatment.

In patients with SVR, CXCL9 levels significantly decreased from baseline to week 12, and further decreased at EOT (mean value 228.4, 153.1 and 52.7 pg/mL at baseline, week 12 and EOT, respectively; baseline vs week12, p=0.039; week 12 vs EOT, p=0.208; Figure 1). In patients without SVR, CXCL9 levels remained high at week 12 but decreased later from week 12 to EOT (mean value 126.2, 136.2 and 103.8 pg/mL at baseline, week 12 and EOT, respectively; baseline vs week12, p=0.754; week 12 vs EOT, p=0.005, Figure 1).

The early CXCL9 levels decline positively correlated with the declines of HBV DNA (r=0.319, p=0.014) and HBsAg levels (r=0.315, p=0.023) at week 12 of PEG-IFN therapy. In contrast, no significant correlations were noted between IP-10 levels decline and declines of viral load or HBsAg levels.

### Correlation between serum ALT and chemokine levels

**Figure 2:**
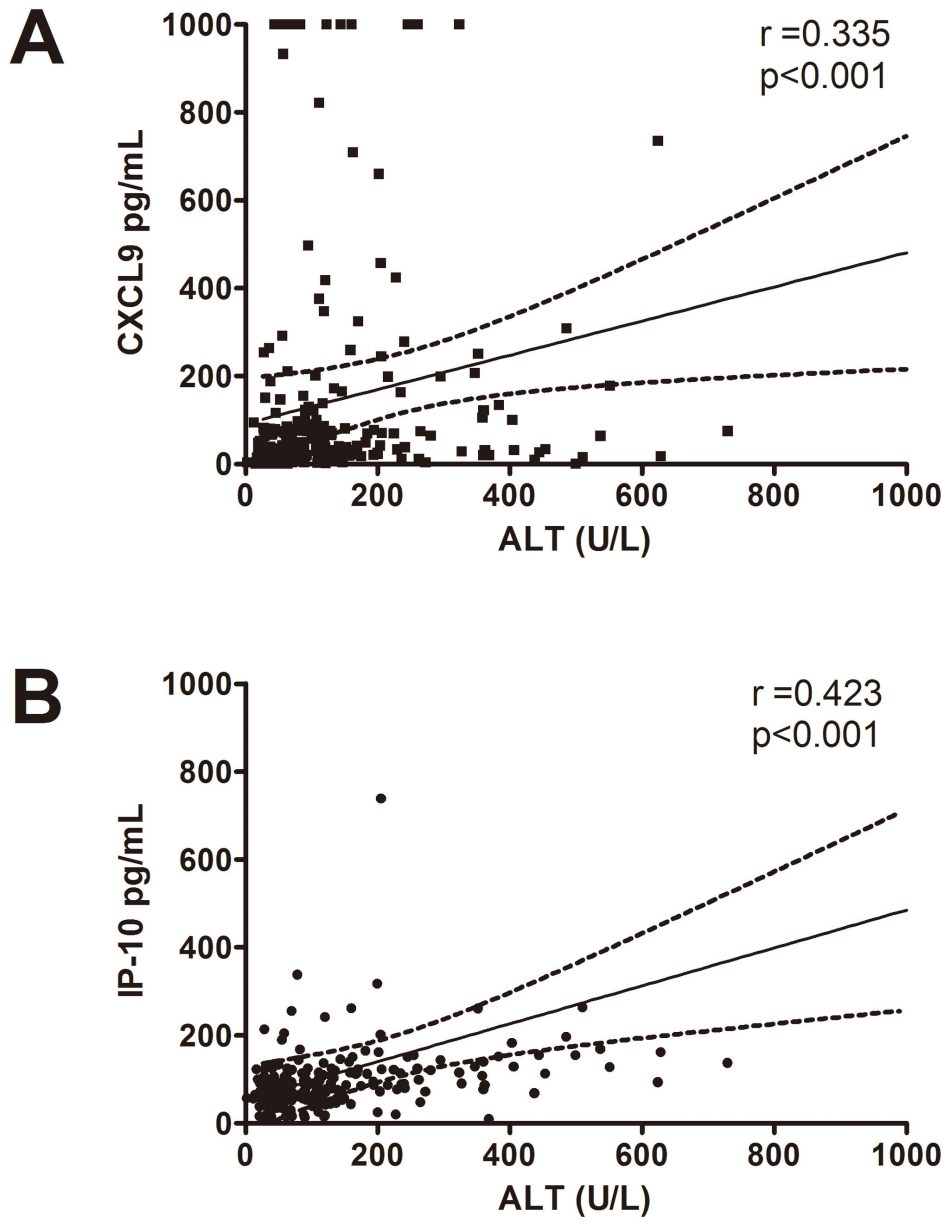
Correlations between serum ALT and chemokine levels. (A) A positive correlation between CXCL9 and ALT; (B). A positive correlation between IP-10 and ALT. Concentrations of serum ALT, CXCL9 and IP-10 levels were collected at each time point during PEG-IFN treatment (overall n=232).

Serum ALT, CXCL9 and IP-10 levels were measured at each time point during PEG-IFN treatment for correlation analysis (overall n=232). As shown in Figure 2, ALT had positive correlation with both serum CXCL9 (r=0.335, p<0.001) and IP-10 levels (r=0.423, p<0.001) by Spearman’s test. Serum CXCL9 and IP-10 levels also had a positive correlation with each other (r=0.353, p<0.001).

### Baseline predictors of SVR

Compared with patients not achieving SVR, patients with SVR had significantly lower HBV DNA, HBsAg levels and higher CXCL9 levels (Table 1). In contrast, HBV genotypes, precore or BCP mutations, serum IP-10, IFN-γ, TGF-β levels and the four IL28B SNPs were not associated with SVR. In univariate analysis, age, HBV viral loads, HBsAg and CXCL9 levels were factors associated with SVR (Table 3). In multivariate analysis, HBV DNA <2.5 x 10^7^ IU/mL and CXCL9 >80 pg/mL were pre-treatment predictors of SVR (Table 3).

**Table 3:** Univariate and multivariate analyses of factors associated with sustained virological response.

		Univariate			Multivariate		
		OR	95% CI	*p*	OR	95% CI	*p*
**Pretreatment predictor**							
Age (years)	>40 vs ≤40	3.360	1.063-10.620	0.039			NS
Sex	male vs female	1.161	0.327-4.118	0.818			NA
HBeAg status	positive vs negative	0.523	0.179-1.528	0.236			NA
Treatment duration	24 vs 48 weeks	0.596	0.198-1.799	0.359			NA
HBV genotype	B vs C	1.238	0.407-3.766	0.707			NA
BCP mutation	mutant vs wild type	0.734	0.229-2.356	0.603			NA
Precore mutation	mutant vs wild type	2.385	0.814-6.989	0.113			NA
*IL28B* polymorphisms							
rs8105790	CC vs CT/TT	-	-	NS*			NA
rs12979860	TT vs CT/TT	-	-	NS*			NA
rs8099917	GG vs GT/TT	-	-	NS*			NA
rs10853728	CC vs CG/GG	1.149	0.355-3.721	0.817			NA
HBV DNA (IU/mL)	>2.5 x 10^7^ vs ≤2.5 x 10^7^	0.062	0.008-0.497	0.009	0.077	0.009-0.654	0.019
HBsAg (IU/mL)	>2000 vs ≤2000	0.240	0.073-0.786	0.018			NS
ALT (U/L)	>400 vs ≤400	1.875	0.403-8.729	0.423			NA
CXCL9 (pg/mL)	>80 vs ≤80	8.200	2.500-26.894	0.001	6.791	1.924-23.967	0.003
IP-10 (pg/mL)	>80 vs ≤80	1.059	0.360-3.116	0.917			NA
IFN-γ (pg/mL)	>40 vs ≤40	0.427	0.124-1.469	0.177			NA
TGF-β (pg/mL)	>1000 vs ≤1000	1.887	0.657-5.433	0.238			NA
**On-treatment predictor (week 12)**							
HBV DNA (IU/mL)	>2000 vs ≤2000	0.154	0.043-0.548	0.004	0.079	0.017-0.377	0.001
HBsAg (IU/mL)	>1000 vs ≤1000	0.142	0.040-0.508	0.003			NS
CXCL9 (pg/mL)	>30 vs ≤30	2.414	0.599-9.791	0.215			NA
IP-10 (pg/mL)	>50 vs ≤50	3.889	0.977-15.473	0.054			NS
IFN-γ (pg/mL)	>50 vs ≤50	0.587	0.140-2.470	0.468			NA
TGF-β (pg/mL)	>1250 vs ≤1250	1.891	0.590-6.056	0.284			NA
HBV DNA decline	>2 Log_10_ vs ≤2 Log_10_	14.400	1.763-117.629	0.013			NS
HBsAg decline	>10% vs ≤10%	-	-	-*	-	-	-*
CXCL9 change	decrease vs increase	2.591	0.715-9.387	0.147			NA
IP-10 change	decrease vs increase	1.049	0.303-3.630	0.940			NA
IFN-γ change	decrease vs increase	1.905	0.340-10.667	0.464			NA
TGF-β change	decrease vs increase	0.961	0.296-3.116	0.947			NA

OR, odds ratio; CI, confidence interval; NA, not adopted; NS, not significant.

* All patients with minor rs8105790, rs12979860 and rs8099917 genotypes did not achieve SVR. None of the patients who did not have HBsAg decline >10% at week 12 achieved SVR.

In HBeAg-positive patients, age, precore mutation, baseline HBV viral loads and CXCL9 levels correlated with SVR in univariate analysis, while HBV DNA <2.5 x 10^7^ IU/mL was the only predictor of SVR in multivariate analysis (Table S1). In HBeAg-negative patients, HBV DNA, HBsAg and CXCL9 levels correlated with SVR in univariate analysis, while CXCL9 >80 pg/mL was the only pre-treatment predictors of SVR in multivariate analysis (Table S2).

### On-treatment predictors of SVR

In univariate analysis, HBV viral load and HBsAg levels, HBV DNA decline >2 Log_10_ and HBsAg decline >10% at week 12 were on-treatment factors associated with SVR (Table 3). None of the patients who did not have HBsAg decline >10% at week 12 achieved SVR. Although significant dynamic changes of IP-10 and IFN-γ levels from baseline to week 12 were noted, none of these factors were associated with SVR. In multivariate analysis, the on-treatment predictors of SVR were HBV DNA <2000 IU/mL and HBsAg decline >10% at week 12 (Table 3).

In HBeAg-positive patients, only HBV DNA decline >2 Log_10_ was the on-treatment predictor SVR in uni- and multi-variate analyses (Table S1). In HBeAg-negative patients, HBsAg levels and HBsAg decline >10% correlated with SVR in univariate analysis, while HBsAg decline >10% was the only on-treatment predictor of SVR in multivariate analysis (Table S2).

### Baseline CXCL9 levels and on-treatment response

We further analyzed the correlation between baseline CXCL9 levels and on-treatment viral suppression, determined by HBV DNA ever less than 20,000 IU/mL during PEG-IFN treatment. The mean baseline CXCL9 levels in patients with or without achieving HBV DNA <20,000 IU/mL during PEG-IFN treatment were 191.6 vs. 68.5 pg/mL, respectively (p=0.004). Among the 28 patients with baseline CXCL9 >80 pg/mL, 24 (85.7%) had ever achieved HBV DNA <20,000 IU/mL, whereas in the 46 patients with baseline CXCL9 <80 pg/mL, only 26 (56.5) had ever achieved HBV DNA <20,000 IU/mL (p=0.019).

### Predictive values of baseline CXCL9 levels on SVR

**Figure 3:**
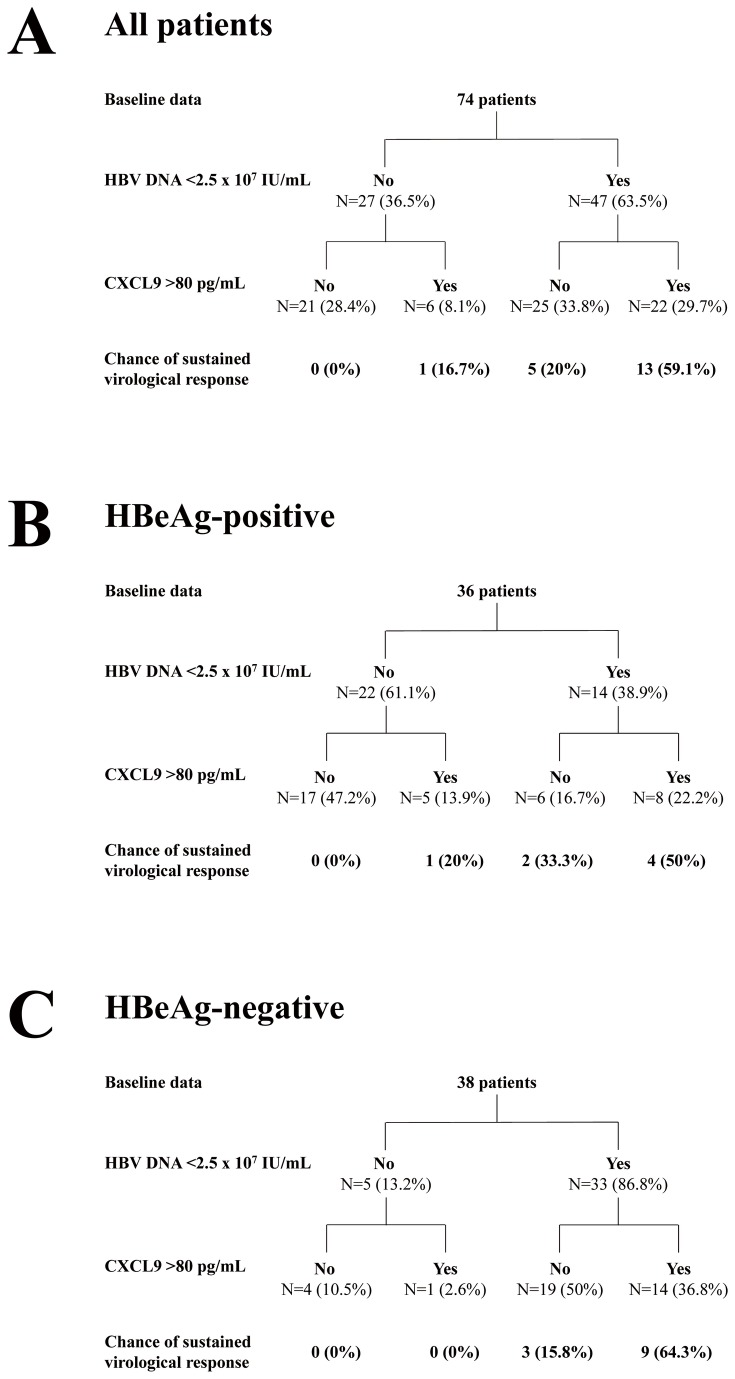
Prediction algorithm based on baseline serum CXCL9 level and HBV viral load. The prediction algorithm applied for all (A), HBeAg-positive (B) and HBeAg-negative CHB patients (C).

In order to identify patients who had higher chance of SVR before PEG-IFN treatment, an algorithm based on combination of baseline serum CXCL9 and HBV DNA levels was established (Figure 3). Patients with baseline high viral loads generally had a low chance of SVR. Among 47 patients with low viral loads, 13 of 22 patients with CXCL9 >80 pg/mL achieved SVR (positive predictive value (PPV)=59.1%), whereas only 5 of 25 (20%) patients with CXCL9 <80 pg/mL had SVR (Figure 3A). The SVR rate was lowest (0%) in patients with baseline low CXCL9 levels but high viral loads (negative predictive value (NPV) = 100%). The predictive value of CXCL9 was not dominant in HBeAg-positive patients (Figure 3B), but the performance of CXCL9 in predicting SVR was good in HBeAg-negative patients with low viral loads (PPV=64.3%, Figure 3C).

## Discussion

Interferon therapy for CHB remains a clinical challenge. Although successful PEG-IFN treatment may induce a durable response and reduce liver-related complications, most of patients could not achieve virological response [10–13], and only a minority of patients choose PEG-IFN as initial antiviral treatment for CHB due to the concern of side effects in Taiwan. Therefore, individualized treatment strategies according to pretreatment and on-treatment parameters should be developed to identify patients with a highest chance of response. However, currently there is no ideal pre-treatment predictor of SVR, especially in HBeAg-negative patients. In this prospective pilot study, we demonstrated the dynamic changes of serum cytokines and chemokines during PEG-IFN therapy and showed that baseline CXCL9 level strongly correlated with SVR in patients with CHB.

In the current study, the HBeAg seroconversion rate and SVR rate in HBeAg-positive patients receiving 24-week PEG-IFN treatment (33.3% and 20%, respectively) were comparable with the results reported in previous studies [11,33,34]. In HBeAg-negative patients, the SVR (31.6%) and HBsAg clearance (5.3%) rates were also consistent with those from the previous reports [35,36].

Declines of serum IP-10 levels have been observed during PEG-IFN therapy in HBeAg-positive CHB patients [37]. In our study, we also observed a significant decline of IP-10 and IFN-γ levels 12 weeks after PEG-IFN treatment, which might reflect a lessened hepatic necro-inflammatory activity after treatment. Although there was no significant change in CXCL9 levels at week 12 in overall patients, patients with SVR had a significant early decline of CXCL9 levels while patients without SVR had a late CXCL9 levels decline (Figure 1). The early CXCL9 decline in patients with SVR might represent an early viral suppression, as supported by the positive correlations between early CXCL9 levels decline and declines of viral load and HBsAg levels. Unfortunately, the early CXCL9 decline could not accurately predict SVR (SVR rate of 35.3% and 17.4% in patients with and without early CXCL9 decline, respectively, p=0.240). Consistent with recent studies showing that HBsAg decline at week 12 strongly correlated with SVR [17,18,38], our data also demonstrated that none of the patients who did not have HBsAg decline >10% at week 12 achieved SVR in this study. HBsAg clearance is a benefit of interferon treatment. It will be interesting to define the relationship between the baseline level of HBsAg and HBsAg clearance, as well as the role of baseline level and dynamic changes of CXCL9 on HBsAg loss. However, during the limited follow-up period of this study, only 2 cases achieved HBsAg clearance. Further large long-term follow-up studies are needed to delineate the role of CXCL9 on HBsAg clearance.

The finding that older patients have higher rates of SVR in univariate analysis might relate to lower viral loads in elder people. Although IL28B SNPs have been shown to predict SVR in patients with chronic hepatitis C and correlate with hepatitis activity in CHB [4,25–27], there was no correlation between IL28B SNPs and SVR in both HBeAg-positive and HBeAg-negative patients. The role of CXCL9 in CHB patients receiving PEG-IFN has not been reported before. CXCL9 and IP-10 were reported to correlate with hepatic injury during hepatic flares in CHB [19]. A strong correlation of serum CXCL9 and IP-10 concentrations with ALT levels was also noted [19]. Consistent with previous report, our results also showed positive correlations between CXCL9, IP-10 and ALT levels. The positive correlations between these parameters may imply that these chemokines have positive impact on the liver inflammation and HBV control, albeit the relevance was not good due to complex interactions between other host and viral factors that contribute the liver inflammation. These findings suggest that both CXCL9 and IP-10 are associated with hepatic necro-inflammation and may be surrogates of the host immune response against HBV infection. IP-10 has been shown to correlate with SVR to IFN-based therapy in patients with HCV infection [20–22]. Sonneveld, et al recently reported that high pretreatment serum IP-10 levels were associated with HBeAg loss after 52 weeks of PEG-IFN therapy in HBeAg-positive patients, albeit the predictive value for combined serological and virological response was less prominent [37]. Different HBV genotypes and duration of PEG-IFN treatment may influence the predictive role of IP-10 in HBeAg-positive patients.

Baseline CXCL9 level significantly correlates not only with SVR, but also with on-treatment viral suppression. This finding supports a stronger host immune response at baseline having a higher chance of SVR under interferon treatment. A recent finding also showed that HBeAg-negative CHB patients with greater baseline HBV-specific CTL responses had a better on-treatment control of HBV replication during PEG-IFN treatment [39].

CXCL9 and IP-10 are chemokines that bind to the cell surface chemokine receptor CXCR3, which is highly expressed on effector T cells and plays an important role in T cell trafficking and function [40,41]. An animal study showed that HBV-specific cytotoxic T lymphocytes (CTLs) induced CXCL9 and IP-10 production and subsequently recruited host inflammatory cells responsible for liver damage [42,43]. Lower levels of CXCL9 and IP-10 correlate with HBV persistence in an animal model [44]. In human study, elevated CXCL9 and IP-10 involve in liver inflammation during hepatitis flares in CHB [19]. Our results showed a positive correlation between CXCL9 and IP-10 levels, representing that both chemokines were induced and participated in immune responses against HBV infection. Although both CXCL9 and IP-10 are ligands of CXCR3, they have distinct properties. IP-10 is induced by a variety of innate stimuli that can further induce IFN-α/β, and is responsible for recruiting type-1 helper (Th1)-type CD4^+^ T cells, effector CD8^+^ T cells and innate-type lymphocytes, such as natural killer (NK) and NKT cells. In contrast, the induction of CXCL9 is restricted to IFN-γ, and its function is predominantly in recruiting CD8^+^ cytotoxic T lymphocytes [41]. The underlying mechanism of CXCL9 level correlates with SVR deserves further study.

The strong correlation of baseline CXCL9 levels with SVR indicates that CXCL9 might serve as a marker to select CHB patients suitable for PEG-IFN therapy, especially in HBeAg-negative cases with low viral loads. In contrast, the SVR rate in patients with high viral loads was low regardless of their baseline CXCL9 levels, suggesting that host antiviral immunity might be overwhelmed by high viral loads.

As a pilot study, the case number was small. It was also due to fewer than 5% of CHB patients in Taiwan had the will to receive PEG-IFN as initial antiviral treatment. Therefore, it is important to identify a pre-treatment marker to predict response to PEG-IFN, not only to encourage patients to receive PEG-IFN treatment but also to save the medical resources for those with a high chance of response. Further large and long-term follow up studies are needed to determine the appropriate cutoff level of CXCL9, and to observe the predictive value of CXCL9 on delayed virological response and HBsAg clearance.

In conclusion, CXCL9 might be used to select appropriate CHB patients to receive PEG-IFN treatment, especially in HBeAg-negative patients with low viral loads.

## Supporting Information

Table S1 **Univariate and multivariate analyses of factors associated with sustained virological response in HBeAg-positive patients (n=36).**
(DOC)

Table S2 **Univariate and multivariate analyses of factors associated with sustained virological response in HBeAg-negative patients (n=38).**
(DOC)
