# Supplementary material for: CXCL9 Associated with Sustained Virological Response in Chronic Hepatitis B Patients Receiving Peginterferon Alfa-2a Therapy: A Pilot Study
Source: PLoS One. 2013 Oct 4;8(10):e76798. doi: 10.1371/journal.pone.0076798 (PMC3790882; doi:10.1371/journal.pone.0076798)
Supplement: Table S1 — Univariate and multivariate analyses of factors associated with sustained virological response in HBeAg-positive patients (n=36). (DOC) [file pone.0076798.s001.doc]

Table S1. Univariate and multivariate analyses of factors associated with sustained virological response in HBeAg-positive patients (n=36)

|  |  | Univariate | | |  | Multivariate | | |
| --- | --- | --- | --- | --- | --- | --- | --- | --- |
|  |  | OR | 95% CI | *p* |  | OR | 95% CI | *p* |
| **Pretreatment predictor** | | | | | | | | |
| Age (years) | >40 vs ≤40 | 7.857 | 1.239-49.834 | 0.029 |  |  |  | NS |
| Sex | male vs female | 1.125 | 0.183-6.935 | 0.899 |  |  |  | NA |
| Treatment duration | 24 vs 48 weeks | 1.250 | 0.122-12.796 | 0.851 |  |  |  | NA |
| HBV genotype | B vs C | 0.941 | 0.177-4.997 | 0.943 |  |  |  | NA |
| BCP mutation | mutant vs wild type | 0.889 | 0.144-5.479 | 0.899 |  |  |  | NA |
| Precore mutation | mutant vs wild type | 7.857 | 1.239-49.834 | 0.029 |  |  |  | NS |
| *IL28B* polymorphisms |  |  |  |  |  |  |  |  |
| rs8105790 | CC vs CT/TT | - | - | NS* |  |  |  | NA |
| rs12979860 | TT vs CT/TT | - | - | NS* |  |  |  | NA |
| rs8099917 | GG vs GT/TT | - | - | NS* |  |  |  | NA |
| rs10853728 | CC vs CG/GG | 0.652 | 0.101-4.231 | 0.654 |  |  |  | NA |
| HBV DNA (IU/mL) | >2.5 x 10^7^ vs ≤2.5 x 10^7^ | 0.063 | 0.007-0.613 | 0.017 |  | 0.063 | 0.007-0.613 | 0.017 |
| HBsAg (IU/mL) | >2000 vs ≤2000 | 0.762 | 0.067-8.726 | 0.827 |  |  |  | NA |
| ALT (U/L) | >400 vs ≤400 | - | - | NS* |  |  |  | NA |
| CXCL9 (pg/mL) | >80 vs ≤80 | 6.562 | 1.052-40.946 | 0.044 |  |  |  | NS |
| IP-10 (pg/mL) | >80 vs ≤80 | 0.952 | 0.153-5.942 | 0.958 |  |  |  | NA |
| IFN-γ (pg/mL) | >40 vs ≤40 | 0.680 | 0.111-4.182 | 0.677 |  |  |  | NA |
| TGF-β (pg/mL) | >1000 vs ≤1000 | 1.778 | 0.333-9.478 | 0.500 |  |  |  | NA |
| **On-treatment predictor (week 12)** | | | | | | | | |
| HBV DNA (IU/mL) | >2000 vs ≤2000 | 0.190 | 0.026-1.416 | 0.105 |  |  |  | NA |
| HBsAg (IU/mL) | >1000 vs ≤1000 | 0.286 | 0.036-2.297 | 0.239 |  |  |  | NA |
| CXCL9 (pg/mL) | >30 vs ≤30 | 2.500 | 0.253-24.719 | 0.433 |  |  |  | NA |
| IP-10 (pg/mL) | >50 vs ≤50 | 1.857 | 0.290-11.902 | 0.514 |  |  |  | NA |
| IFN-γ (pg/mL) | >50 vs ≤50 | 0.571 | 0.054-6.079 | 0.643 |  |  |  | NA |
| TGF-β (pg/mL) | >1250 vs ≤1250 | 2.250 | 0.317-15.973 | 0.417 |  |  |  | NA |
| HBV DNA decline | >2 Log_10_ vs ≤2 Log_10_ | - | - | -* |  | - | - | -* |
| HBsAg decline | >10% vs ≤10% | - | - | NS* |  |  |  | NA |
| CXCL9 change | decrease vs increase | 3.929 | 0.399-38.704 | 0.241 |  |  |  | NA |
| IP-10 change | decrease vs increase | - | - | NS* |  |  |  | NA |
| IFN-γ change | decrease vs increase | - | - | NS* |  |  |  | NA |
| TGF-β change | decrease vs increase | 0.545 | 0.083-3.590 | 0.528 |  |  |  | NA |

OR, odds ratio; CI, confidence interval; NA, not adopted; NS, not significant.

* All patients with minor rs8105790, rs12979860, rs8099917 genotypes and ALT >400 U/L did not achieve SVR. None of the patients who did not have HBV DNA decline >2 Log_10_, HBsAg decline >10%, IP-10 decrease or IFN-γ decrease at week 12 achieved SVR.
