# Supplementary material for: CXCL9 Associated with Sustained Virological Response in Chronic Hepatitis B Patients Receiving Peginterferon Alfa-2a Therapy: A Pilot Study
Source: PLoS One. 2013 Oct 4;8(10):e76798. doi: 10.1371/journal.pone.0076798 (PMC3790882; doi:10.1371/journal.pone.0076798)
Supplement: Table S2 — Univariate and multivariate analyses of factors associated with sustained virological response in HBeAg-negative patients (n=38). (DOC) [file pone.0076798.s002.doc]

Table S2. Univariate and multivariate analyses of factors associated with sustained virological response in HBeAg-negative patients (n=38)

|  |  | Univariate | | |  | Multivariate | | |
| --- | --- | --- | --- | --- | --- | --- | --- | --- |
|  |  | OR | 95% CI | *p* |  | OR | 95% CI | *p* |
| **Pretreatment predictor** | | | | | | | | |
| Age (years) | >40 vs ≤40 | 1.333 | 0.283-6.279 | 0.716 |  |  |  | NA |
| Sex | male vs female | 0.909 | 0.142-5.809 | 0.920 |  |  |  | NA |
| HBV genotype | B vs C | 1.333 | 0.283-6.279 | 0.716 |  |  |  | NA |
| BCP mutation | mutant vs wild type | 0.630 | 0.135-2.926 | 0.555 |  |  |  | NA |
| Precore mutation | mutant vs wild type | 0.875 | 0.275-3.525 | 0.851 |  |  |  | NA |
| *IL28B* polymorphisms |  |  |  |  |  |  |  |  |
| rs8105790 | CC vs CT/TT | - | - | NS* |  |  |  | NA |
| rs12979860 | TT vs CT/TT | - | - | NS* |  |  |  | NA |
| rs8099917 | GG vs GT/TT | - | - | NS* |  |  |  | NA |
| rs10853728 | CC vs CG/GG | 1.875 | 0.407-8.633 | 0.420 |  |  |  | NA |
| HBV DNA (IU/mL) | >2.5 x 10^7^ vs ≤2.5 x 10^7^ | - | - | -* |  |  |  | NS |
| HBsAg (IU/mL) | >2000 vs ≤2000 | 0.159 | 0.028-0.899 | 0.037 |  |  |  | NS |
| ALT (U/L) | >400 vs ≤400 | 4.000 | 0.571-28.011 | 0.163 |  |  |  | NA |
| CXCL9 (pg/mL) | >80 vs ≤80 | 10.000 | 2.032-49.215 | 0.005 |  | 10.000 | 2.032-49.215 | 0.005 |
| IP-10 (pg/mL) | >80 vs ≤80 | 1.400 | 0.352-5.572 | 0.633 |  |  |  | NA |
| IFN-γ (pg/mL) | >40 vs ≤40 | 0.300 | 0.054-1.669 | 0.169 |  |  |  | NA |
| TGF-β (pg/mL) | >1000 vs ≤1000 | 2.250 | 0.552-9.170 | 0.258 |  |  |  | NA |
| **On-treatment predictor (week 12)** | | | | | | | | |
| HBV DNA (IU/mL) | >2000 vs ≤2000 | 0.182 | 0.032-1.039 | 0.055 |  |  |  | NS |
| HBsAg (IU/mL) | >1000 vs ≤1000 | 0.121 | 0.020-0.753 | 0.024 |  | 0.083 | 0.006-1.069 | 0.056 |
| CXCL9 (pg/mL) | >30 vs ≤30 | 2.455 | 0.395-15.252 | 0.335 |  |  |  | NA |
| IP-10 (pg/mL) | >50 vs ≤50 | 7.000 | 0.722-67.840 | 0.093 |  |  |  | NS |
| IFN-γ (pg/mL) | >50 vs ≤50 | 0.667 | 0.099-4.478 | 0.667 |  |  |  | NA |
| TGF-β (pg/mL) | >1250 vs ≤1250 | 1.714 | 0.371-7.918 | 0.490 |  |  |  | NA |
| HBV DNA decline | >2 Log_10_ vs ≤2 Log_10_ | 5.714 | 0.613-53.229 | 0.126 |  |  |  | NA |
| HBsAg decline | >10% vs ≤10% | - | - | -* |  | - | - | -* |
| CXCL9 change | decrease vs increase | 2.333 | 0.439-12.398 | 0.320 |  |  |  | NA |
| IP-10 change | decrease vs increase | 1.000 | 0.206-4.856 | 1.000 |  |  |  | NA |
| IFN-γ change | decrease vs increase | 0.857 | 0.098-7.510 | 0.889 |  |  |  | NA |
| TGF-β change | decrease vs increase | 1.500 | 0.299-7.531 | 0.622 |  |  |  | NA |

OR, odds ratio; CI, confidence interval; NA, not adopted; NS, not significant.

* All patients with minor rs8105790, rs12979860 and rs8099917 genotypes did not achieve SVR. None of the patients who did not have baseline HBV DNA ≤2.5 x 10^7^ and HBsAg decline >10% at week 12 achieved SVR.
